# The Association of Histological Signs of Plaque Instability with Low eGFR, Higher Neutrophil-to-Lymphocyte Ratio, and Lower Serum MCP-1 Levels in Carotid Endarterectomy Patients—A Single-Center, Prospective Cohort Study

**DOI:** 10.3390/life15071008

**Published:** 2025-06-25

**Authors:** Ioan Alexandru Balmos, Adina Huțanu, Adrian Vasile Muresan, Előd Ernő Nagy, Klara Brinzaniuc, Gyopár Beáta Molnár, Rita Szodorai, Emőke Horváth

**Affiliations:** 1 Department of Anatomy, George Emil Palade University of Medicine, Pharmacy, Science, and Technology of Targu Mures, 540142 Targu Mures, Romania; ioan.balmos@umfst.ro (I.A.B.); klara.brinzaniuc@umfst.ro (K.B.); 2 Vascular Surgery Clinic, County Emergency Clinical Hospital of Targu Mures, 540136 Targu Mures, Romania; adrian.muresan@umfst.ro; 3 Center for Advanced Medical and Pharmaceutical Research, George Emil Palade University of Medicine, Pharmacy, Science, and Technology of Targu Mures, 540142 Targu Mures, Romania; 4 Department of Laboratory Medicine, George Emil Palade University of Medicine, Pharmacy, Science, and Technology of Targu Mures, 540142 Targu Mures, Romania; 5 M3 Department of Surgery, George Emil Palade University of Medicine, Pharmacy, Science, and Technology of Targu Mures, 540142 Targu Mures, Romania; 6 Department of Biochemistry and Environmental Chemistry, George Emil Palade University of Medicine, Pharmacy, Science, and Technology of Targu Mures, 540142 Targu Mures, Romania; elod.nagy@umfst.ro (E.E.N.); gyopar.molnar@umfst.ro (G.B.M.); 7 Laboratory of Medical Analysis, Clinical County Hospital Mures, 540394 Targu Mures, Romania; 8 Doctoral School of Medicine and Pharmacy, I.O.S.U.D., George Emil Palade University of Medicine, Pharmacy, Science, and Technology of Targu Mures, 540142 Targu Mures, Romania; 9 Pathology Service, County Emergency Clinical Hospital of Targu Mures, 540136 Targu Mures, Romania; rita.szodorai@umfst.ro (R.S.); emoke.horvath@umfst.ro (E.H.); 10 Department of Pathology, Faculty of Medicine, George Emil Palade University of Medicine, Pharmacy, Science and Technology of Targu Mures, 540142 Targu Mures, Romania

**Keywords:** plaque vulnerability, intraplaque hemorrhage, eGFR, biomarkers, carotid stenosis, endarterectomy

## Abstract

**Background**: Histological signs of carotid atheromatous plaque vulnerability, such as hemorrhage, neovascularization, atherothrombosis, and ulceration, develop against an unstable biological background. Declining renal function contributes to atherosclerotic progression and worsens cardiovascular outcomes. **Methods**: In a single-center prospective cohort study, we studied 41 endarterectomized patients with severe carotid atherosclerosis. The histological samples were stained with H&E to assess morphology and immunohistochemically labeled with antibodies for CRP and MMP-9 proteins. Complete blood count, the presence of serum biomarkers hsCRP, oxLDL, MCP-1, and MMP-9, and the level of eGFR were determined. **Results**: Twenty-eight patients with complicated plaques had significantly lower eGFR values: 79.5 (24–110) vs. 94 (69–114) (*p* = 0.004). Patients with eGFR > 90 mL/min/1.73m^2^ had a higher incidence of intraplaque hemorrhage and histologic complications of any cause (*p* = 0.012 and *p* = 0.003). Patients with bleeding and ulceration from the carotid plaque had a higher neutrophil/lymphocyte ratio. Significantly lower levels of MCP-1 were found in the serum of patients with massive inflammatory infiltrate of the carotid plaques, while serum levels of biomarkers like hsCRP, MMP-9, and oxLDL did not show differences in cases with plaque vulnerability. **Conclusions**: Signs of plaque vulnerability are associated with reduced renal function, a higher neutrophil/lymphocyte ratio, and lower serum levels of MCP-1 in advanced carotid artery stenosis disease.

## 1. Introduction

Aging-related changes are observed in all organs, particularly in the cardiovascular system. It is well established that these changes primarily affect the arterial wall. Pathophysiological alterations within the arterial wall in older individuals have been identified as the primary drivers of increased arterial stiffness and the development of atherosclerosis [1]. In most cases, this results in severe narrowing of the artery, or, in the case of unstable plaques, leads to the rupture of the artery and subsequent cardiovascular events [2]. In the carotid artery, the main blood supply to the brain, the rupture of an atherosclerotic unstable plaque at the level of the common or internal carotid artery can determine the occurrence of stroke [3], the second leading cause of death and the leading cause of disability worldwide [4]. In addition, the presence of carotid artery disease may contribute to cognitive decline, highlighting the importance of understanding carotid health in the context of both stroke risk and overall neurological function [5]. It has been accepted for several decades that atherosclerosis is a chronic inflammatory disease, caused by dyslipidemia and mediated by innate and acquired immunity through inflammatory factors produced by cells [6], and the morphology of atherosclerotic lesions in the carotid artery is linked with increased serum levels of inflammatory markers, including hs-CRP and MMP-9 [7,8].

In the clinical context, the main risk factors for stroke are hypertension, hyperlipidemia, atrial fibrillation, diabetes mellitus, smoking, and obesity [9]; however, the close relationship between chronic kidney disease (affecting as many as one-third of the population over the age of 75 years) and peripheral atherosclerosis (coronary atherosclerosis, peripheral arterial disease, and carotid atherosclerosis) is well known and is considered an important risk factor for stroke [10]. At the same time, mounting clinical and basic research evidence supports the existence of a bidirectional brain–kidney crosstalk following stroke. In this context, it is possible that a stroke can induce renal dysfunction, which may manifest as acute kidney injury (AKI) or chronic kidney disease (CKD) [11]. The findings of cohort studies and clinical trials suggest there is a positive correlation between a reduced glomerular filtration rate and an increased risk of stroke, with a reported increase of approximately 40% [12]. Weiner’s meta-analysis, based on large, longitudinal population-based studies, showed that people with an eGFR of <60 mL/min/1.73 m^2^ had an incidence rate of 10.3 stroke events per 1000 person-years, compared with 3.4 events per 1000 person-years in those with an eGFR of >60 mL/min/1.73 m^2^ [13].

The literature on this subject is inconclusive, with conflicting evidence regarding the role of CKD, specifically low estimated glomerular filtration rate (eGFR), as an independent risk factor for stroke. For instance, Sandsmark et al. have demonstrated that proteinuria and albuminuria are better predictors of stroke risk in patients with chronic kidney disease compared to eGFR [14].

On the other hand, complicated plaques predict vascular events. Studies have linked MRI or CT-imaging and the histological evidence of ulcerative, hemorrhagic, and necrotic lipid-core-containing plaques in carotid arteries, with an increased risk of acute or future stroke in the ipsilateral zones of the brain [15,16,17].

This present clinical–pathological study aims to correlate renal function in patients with severe carotid stenosis with various biological and histological indicators of plaque vulnerability, using a complex clinical–pathological and immunohistochemical approach.

## 2. Materials and Methods

### 2.1. Patient Data

A total of 52 patients with advanced atherosclerosis of the carotid artery were selected for clinical–pathological study according to strict criteria. Individuals with a second stenotic lesion in the intracranial segment of the internal carotid artery, those who experienced restenosis after CEA of the same artery, patients with near occlusion of the carotid artery (defined as severe stenosis with collapse of the distal vessel), and individuals with hematological disorders were excluded from the study, as were patients with improper histological material or with serum/plasma hemolysis. As a result, only 41 patients with severe carotid stenosis (>70%) who were candidates for carotid endarterectomy were enrolled. The level and degree of stenosis were determined by imaging techniques (CT angiography and Doppler ultrasound). Indication for carotid endarterectomy (CEA) was based on a clinician’s judgment, performed according to the European Society for Vascular Surgery and European Stroke Organization guidelines [18]. Clinical data, histopathology report, complete laboratory tests, and written informed consent of enrollment in the study are available for all patients. Demographic information (age and sex) and clinical data (neurological symptoms at the time of admission, history of hypertension, diabetes mellitus, hypercholesterolemia [19,20], previous stroke/transient ischemic attack history [21], and chronic kidney disease (CKD) [22]) were retrieved from the hospital’s computerized database. Body mass index (BMI) was calculated for each patient, who were categorized as lean, normal weight, overweight, and obese for BMI <18.5, 18.5–23.9, 24–27.9, and ≥28 kg/m^2^, respectively. Absolute counts of lymphocytes, neutrophils, and monocytes were obtained from the complete blood count (CBC) data. The values obtained have been reported and compared to the normal range of neutrophils (1.5–8.00 × 10^3^/µL), lymphocytes (1.00–4.0 × 10^3^/µL), and monocytes (0.2–1 × 10^3^/µL) in a healthy adult [23].

The neutrophil/lymphocyte ratio (NLR), lymphocyte/monocyte ratio (LMR), and systemic inflammatory response index (SIRI), calculated as neutrophil count × monocyte/lymphocyte count, were evaluated as possible prognostic predictors in patients with acute ischemic stroke. Concurrently, the eGFR (estimated glomerular filtration rate) was determined based on creatinine levels in accordance with international recommendations [23]. Based on the value of the eGFR, the patients were divided into three groups. Group A (G1) included patients with eGFR >90 mL/min/1.73 m^2^ (normal or high); Group B (G2) included patients with eGFR <90, but ≥60 mL/min/1.73 m^2^ (mildly decreased); and Group C (G3, G4 and G5) included patients with eGFR <60 mL/min/1.73 m^2^ (moderately/severely reduced).

### 2.2. Study of Circulating Biomarkers

Blood samples were collected in EDTA tubes for MMP-9 (matrix metalloproteinase-9) determination and in serum separator tubes (SSTs) for MCP-1 (monocyte chemoattractant protein-1) and oxLDL (oxidized forms of human low-density lipoprotein) quantification before the endarterectomy procedure. After the clothing time for SST, samples were centrifuged at 1500 g, aliquoted, and stored at −80 degrees until the end of the experiment. All samples were analyzed in the same run using the ELISA protocol to avoid biased results due to different working conditions.

In terms of systemic inflammatory markers, the level of hsCRP (high-sensitivity C-reactive protein) was measured using the nephelometric method available on BNProspec Siemens. The principle for hsCRP quantification implied a reaction between serum samples and polystyrene particles coated with anti-CRP monoclonal antibodies. Depending on hsCRP concentration in the sample, the immune complexes formed during the reaction’s scattering of light; the intensity of the scattered light was proportional to the concentration of the hsCRP in the samples.

The response of the monocytes to the chemotactic stimuli was assessed by measuring the serum levels of MCP-1, one of the major chemokines involved in macrophage migration into the periphery, including the vascular endothelium. The MCP-1 concentration was measured using a sandwich ELISA protocol on an automated ELISA Dynex DSX (Dynex Technologies, Chantilly, VA, USA). The sandwich ELISA principle involves two antibodies with MCP-1 specificity: the capture antibody pre-coated onto the bottom of the 96-well plate, and the detection antibody conjugated with biotin. Concomitant with the samples, seven standards with known MCP-1 concentrations were added to the plate to assess sample concentrations on the calibration curves. The measuring range for the MCP-1 kit was between 15.62 and 10,000 pg/mL with a sensitivity of 9.375 pg/mL (Wuhan Fine Biotech Co., Ltd., Wuhan, China). The concentration of the MCP-1 was measured in serum; after an initial 2-fold dilution, the results were adjusted for the dilution factor. For MMP-9, plasma was the matrix of choice; it was diluted 20-fold in our experiment. The same ELISA sandwich protocol was applied for MMP-9 quantification, with a measuring interval ranging from 0.313 to 20 ng/mL and a sensitivity of 0.188 ng/mL. Both capture and detection monoclonal antibodies had excellent specificity for MMP-9 (Wuhan Fine Biotech Co., Ltd., Wuhan, China). After interpolation on the calibration curve, the results were multiplied by the dilution factor for the final concentration.

For oxLDL, the serum samples were diluted twice with sample diluent provided in the kit (Wuhan Fine Biotech Co., Ltd., Wuhan, China). A sandwich ELISA protocol was used for oxLDL measurement, with a measuring range between 3.125 and 200 ng/mL and a sensitivity of 1.875 ng/mL.

In all three ELISA tests, the performance characteristics were <8% and <10% for intra-assay and inter-assay precision, respectively.

### 2.3. Carotid Specimens: Histological Processing

The vascular surgeon excised the carotid plaque specimens using the standard surgical technique detailed in a previously published article [24]. They were immediately fixed in 10% neutral buffered formalin and sent for histopathological analysis. All fragments from each patient were embedded in paraffin blocks and processed according to the standard methodology. Those with calcified or ossified foci were previously decalcified in the ethylene-diamine-tetra-acetic acid (EDTA) solution, with pH 7. Morphological features of carotid plaques were examined in 4–5 µm sections stained with hematoxylin and eosin (H&E) by two pathologists blinded to the patient’s clinical and laboratory data. Consecutive histological sections were prepared for immunohistochemistry.

The morphological characteristics of the plaques were assessed. Those related to plaque vulnerability and plaque complications were highlighted, with each scored as being present or absent: microcalcification or macrocalcification, based on nodules of <50 and ≥50 µm; large lipid core (cellular detritus predominated in its structure, along with macrophages with foamy cytoplasm and cholesterol crystals); intraplaque hemorrhage and fibrous cap damage (ulceration), with or without parietal thrombus fragments; neovascularization (the presence or absence of new vessels within the lipid core); and inflammatory cell invasion (macrophages and lymphocytes). All these morphological alterations were defined based on established criteria drawn from the existing literature and were previously documented in extant publications [25,26]. After determining the presence of inflammatory cells by visual assessment alone, we focused on the number of mononuclear elements. Grading was based on the number of cells around the plaque on hematoxylin-eosin-stained (H&E) sections examined with the Objective 10. Thus, we considered grade 0 as indicating the absence of inflammatory cells or their presence in numbers less than 25. We thought grade 1 was an inflammatory infiltrate of low to moderate severity, with cell counts between 25 and 50. If there were more than 50 mononuclear cells, the inflammation was considered grade 2. Based on the grades, two groups were defined: in the INF− group, the plaques with grade 0 were included. and in the INF+ group, the plaques with grades 1 and 2 were included.

### 2.4. Investigation of the CRP and MMP-9 Expression Within the Atherosclerotic Plaque by Immunohistochemistry

**Figure 1:**
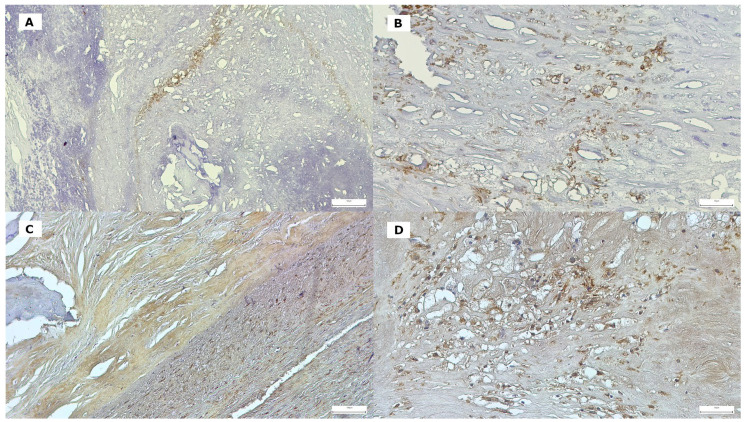
Local synthesis of CRP and MMP-9 by inflammatory cells and vascular smooth muscle cells. Immunohistochemical visualization (3,3′-diaminobenzidine chromogen) of MMP-9 and CRP-labeled cellular and extracellular structures in atherosclerotic plaque. MMP-9 is highly expressed in inflammatory cells (lymphocytes and monocytes) surrounding the lipid core (**A**), but is also detected in the macrophages in the microenvironment of the lipid core (**B**). In the plaque, CRP is also synthesized in vascular smooth cells VSMCs, (**C**) along with macrophages, and various mononuclear elements (**D**).

The local synthesis of CRP and MMP-9 by inflammatory cells and vascular smooth muscle cells (VSMCs) was examined by immunohistochemistry (using a manual technique). Immunohistochemical staining was performed using rabbit monoclonal anti-C-reactive protein antibodies [Y284] (used to detect splice isoform two as well as isoform 1), at a dilution of 1/100, purchased from Abcam, Cambridge, UK, and MMP-9 (rabbit polyclonal antibody, PR066, dilution 1/50), purchased from Diagnostic BioSystem (Pleasanton, CA, USA), in combination with EnVision FLEX/HRP (Agilent, Dako, Santa Clara, CA, USA) as the secondary antibody and 3,3′-diaminobenzidine chromogen (DAB), respectively. These steps were in accordance with the manufacturer’s instructions. Cell nuclei were counterstained with hematoxylin. For the negative control, normal serum was substituted for the primary antibody. Formalin-fixed paraffin-embedded human normal liver tissue was used as the CRP-positive control, and human colon carcinoma tissue was used as the MMP9-positive control. Cytoplasmic immunoreactivity was considered positive for both antigens (Figure 1).

### 2.5. Quantitative Digital Image Analysis: Digital Morphometry

**Figure 2:**
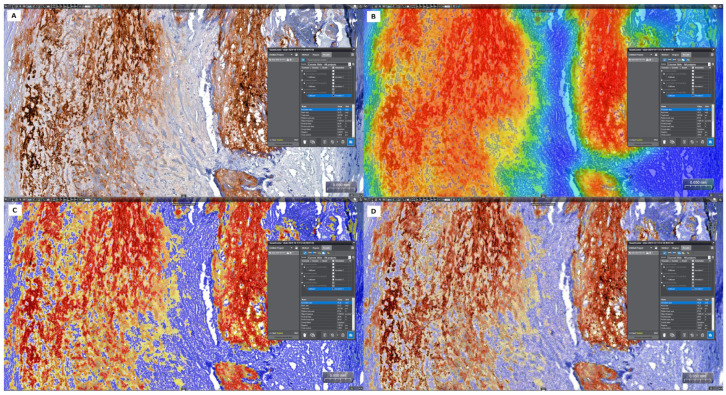
The scanned plaque areas corresponding to the CRP-positive signals were considered for analysis (**A**). Segmentation on anti-CRP/DAB immunolabelled tissue section, followed by annotation of the region of interest. (**B**–**D**). The application of the algorithm to measure the thresholded area and calculate the H-score.

The in situ accumulation of CRP and MMP-9, associated with inflammatory cells, was quantified by digital morphometry. The Panoramic Scan System (3D Histech Ltd., Budapest, Hungary) was used to digitize these ninety-two slides. Using the Panoramic Viewer program, one tissue section was manually annotated from each case, corresponding to the atherosclerotic plaque showing signs of vulnerability. Within the selected annotations, the Pattern Quant module of the SlideViewer-Quant Center version 2.8.0 (3D Histech, Budapest, Hungary) was trained to automatically differentiate between immunostained cell structures (cytoplasm of different inflammatory cells, endothelial cells, and vascular smooth muscle cells), the background, and counterstained tissue. The segmented areas were measured in µm^2^, and the relative mask area (percentage of positive area in the annotation area) was noted from the results (Figure 2). For each case, the H-score was also calculated based on the intensity of the immunohistochemical reaction (1 × percentage of weak staining + (2 × percentage of moderate staining) + (3 × percentage of strong staining), which quantifies biomarker expression of whole-slide-scanned IHC images, providing a dynamic range with which to quantify biomarker abundance.

### 2.6. Statistical Analysis

Categorical variables and transformed continuous variables were assessed for absolute and relative distribution frequency. Analysis of 2 × 2 or 3 × 2 contingency tables was performed with Fisher’s exact test and the Pearson χ^2^ test. Nonlinear logistic regression models were used for the prediction of ulceration and atherothrombosis. In all tests, *p*-values ≤ 0.05 were considered statistically significant. Data processing was performed using Microsoft Excel 2016 (Microsoft Corporation, Redmond, WA, USA) and GraphPad Prism 9.5.1 (GraphPad Software LLC., San Diego, CA, USA).

This study was conducted according to the principles of the Helsinki Declaration. It was approved by the Ethical Committee of the County Emergency Clinical Hospital of Targu Mures, Romania (no. Ad. 1324/26.01.2023) and the Ethical Committee of the George Emil Palade University of Medicine, Pharmacy, Science, and Technology of Targu Mures, Romania (no. 2095/15.02.2023).

## 3. Results

### 3.1. Study Group Characteristics

We included forty-one patients enrolled with advanced carotid artery stenosis (grade 70–95%) who underwent carotid endarterectomy between February 2023 and January 2024 at the Vascular Surgery Clinics of County Emergency Clinical Hospital of Targu Mures. The median age of the study group was 67 (min–max 42–80), with marked male predominance (70.73%). In terms of comorbidities, hypertension (HT) was the most common (95.12%), followed by dyslipidemia (92.68%) and ischemic heart disease (IHD) (82.92%). Polyvascular disease (carotid atherosclerosis with concomitant coronary or peripheral arterial bed involvement) characterized many of the cohort’s subjects (85.36%). The group’s median body mass index (BMI) was 28.37 (20–47.26). Despite the significant number of patients with dyslipidemia, only three patients were classified as displaying grades 2 or 3 of obesity. The degree of carotid stenosis varied from 70% to 95%, with a median of 83.47%. In the study’s population, 16 (39.02%) were smokers. Twenty-six subjects (63.41%) had a history of stroke, and 21.95% of patients met the chronic kidney disease (CKD) criteria according to the KDIGO 2024 Clinical Practice Guideline [22]. The eGFR analysis revealed that 18 patients (43.9%) fell into Group A (above 90 mL/min/1.73 m^2^), 14 patients (34.14%) demonstrated a mild decrease (Group B), and 9 patients (21.95%) exhibited low values (below 60 mL/min/1.73 m^2^, Group C). Peripheral blood test count results showed no significant differences among recruited patients’ absolute neutrophil count, lymphocyte count, and monocyte count. There were significant variations among patients in the neutrophil-to-lymphocyte ratio (NLR), lymphocyte-to-monocyte ratio (LMR), and systemic inflammatory response index (SIRI). Analysis showed that the median concentration of hsCRP was above the reference value (>1.0 mg/L), while the median value for MCP-1 was 235.09 pg/mL, higher than that in healthy individuals [27]. The serum level of oxLDL was within the normal range of 10–170 ng/mL [28]; however, we were not able to estimate the MMP-9 levels in conjunction with the reference intervals since various intervals are mentioned in the literature and the intervals vary according to manufacturer and sample matrix (Table 1).

### 3.2. Histological Features of Plaque Vulnerability in Advanced Carotid Stenosis

**Figure 3:**
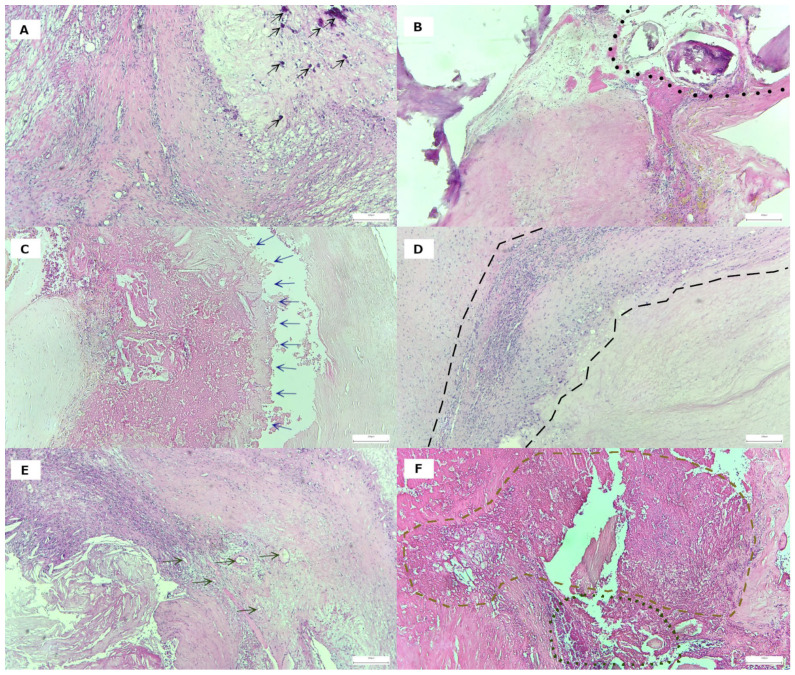
The morphological characteristics of plaque vulnerability and plaque complications per H&E stain (representative section): (**A**) microcalcification (black arrow) or (**B**) macrocalcification (delimited by black dots—based on nodules of <50 and ≥50 μm); (**C**) large lipid core (pointed to by blue arrows—cellular detritus predominant in its structure, together with macrophages with foamy cytoplasm and cholesterol crystals); (**D**) intraplaque inflammatory cell population (delimited by black dashed lines); (**E**) neovascularisation (gray arrows—the presence of new vessels within the lipid core); and (**F**) intraplaque hemorrhage (delimited by light brown dashed lines) and fibrous cap damage (ulceration—delimited by green dots).

First, plaque architecture and histological features were examined by light microscopy, and characteristic signs of plaque vulnerability were reported for each sample. All plaques examined showed signs of calcification of different types. Concerning the calcification patterns, solitaire microcalcification (i.e., numerous scattered small mineral foci forming a calcification front within fibrosis) was present alone in only 12.2% of cases (Figure 3A). All the other instances (87.8%) showed various forms of macrocalcification (with a nodular aspect or extensive (confluent) calcification with or without osteoid metaplasia) (Figure 3B). A lipid-rich, large necrotic core (Figure 3C) was detected in 43.9% of plaques. A mononuclear inflammatory infiltrate (macrophages and lymphocytes), surrounding the lipid core, was found in 32 cases (78.05%), of which 17 cases (41.46%) were grade 1 (Figure 3D) and 15 cases (36.58%) were grade 2 (Figure 3E). Intraplaque hemorrhage was present in 65.85% of cases. Many plaques (85.37%) showed neovascularization due to the proliferation of small, thin-walled microvessels with collapsed lumens (Figure 3F). In seven cases, ulcerated plaques with irregular and discontinuous fibrous caps led to thrombus formation (Figure 3F). Only 13 cases purely displayed stenosing (COMPL− group); the rest of them (28) were associated with various complications (COMPL+ group). The most frequent association seen among these was between intraplaque hemorrhage and neovascularization (22 cases). We identified atherothrombosis in 5 cases and histological signs of ulceration in 4 cases.

Among the histological features of the plaques, there was a predominance of cases with inflammatory infiltrate (78.05%) and neovascularization around the plaque (85.37%). We compared the two features and found that the presence of an inflammatory infiltrate was significantly associated with the proliferation of newly formed vessels (*p* = 0.015).

In terms of biological variables, only eGFR showed a significant difference between the two groups mentioned above, with patients with complicated plaque having a significantly lower eGFR than patients with uncomplicated plaque (*p* = 0.004).

### 3.3. Analysis of In Situ Accumulation of MMP-9 and CRP by Immunohistochemistry and Digital Morphometry

#### 3.3.1. Analysis of the Carotid Plaques with and Without Complications

The local synthesis of CRP and MMP-9 by inflammatory cells and VSMC was quantitatively analyzed by digital morphometry, being performed separately for plaque with different complications (COMPL+ group) and without complications (COMPL− group), in parallel with the determination of the H-score for both biomarkers. The statistical analysis was focused on investigating the correlations between circulating and in situ markers for all characteristics of the study group. Patients with complicated and uncomplicated plaque did not significantly differ in respect to preoperative characteristics—no significant disparities were observed in the incidence of previous stroke history, diabetes, hypertension, or polyvascular involvement.

The main results for demographic data, plaque characteristics, and peripheral biomarkers, in conjunction with complications, are detailed in Table 2.

#### 3.3.2. Analysis of the Carotid Plaques with and Without Inflammatory Infiltrate

We compared 32 cases with medium-grade or strong mononuclear cell infiltrates with a subgroup of 9 plaques showing no inflammatory cell population. The distribution of cases in terms of signs of vulnerability demonstrated no statistically significant bias between the presence of intraplaque inflammatory infiltrate (INF+) and the absence of intraplaque inflammatory infiltrate (INF−). Neither the MMP-9-positive area and H-score nor CRP-positive area and H-score showed significant differences between the INF− and INF+ groups. In the two groups above, we only identified a correlation between the low serum levels of the MCP-1 and the plaque accumulation of moderately/highly inflammatory infiltrates from the circulating biomarkers presented previously, as is evidenced in Table 3.

However, in terms of the correlation of circulating and intraplaque inflammatory markers, we observed a positive association between CRP score and absolute neutrophil count (*p* = 0.042), MMP-9-positive surface area and absolute lymphocyte count (*p* = 0.0005), and SIRI (*p* = 0.023).

### 3.4. Comparative Analysis of Biological Characteristics Associated with Histological Evidence of Hemorrhagic and Any Cause Complicated Plaques

As we found no differences between biological variables in the COMPL+ vs. COMPL− group except for eGFR, we analyzed each sign of plaque vulnerability (microcalcification, intraplaque inflammatory infiltrate, neovascularization, large necrotic lipid core) and pre-existing complications (intraplaque hemorrhage, ulceration, atherothrombosis) separately.

Following the classification of samples according to distinct histologic changes, a positive correlation was observed between the serum MCP-1 level and the presence of the intraplaque mononuclear infiltrate, yet with lower MCP-1 serum values obtained for the INF+ group compared to INF− group (*p* = 0.008). A positive correlation was also observed between the neutrophil-to-lymphocyte ratio (NLR), intraplaque hemorrhage (*p* = 0.039), and plaque ulceration (*p* = 0.025). The latter also correlates with the amount of matrix metalloproteinase-9 (MMP-9) accumulated in the plaque (*p* = 0.019), as several ulcerative plaques expressed higher amounts of MMP-9. Calcification was present in these carotid plaques, and the most calcified lesions, osteoid metaplasia, were present in samples with high degrees of hsCRP expression. These values are presented in Supplementary Table S1.

In the subgroup with hemorrhagic plaques, the neutrophil/lymphocyte ratio was increased (2.8 ± 0.2 vs. 2.2 ± 0.2, *p* = 0.039) and the CRP-positive area was larger, with borderline significance (29.6 ± 6.3 vs. 13.7 ± 6.4, *p* = 0.05).

In view of the statistically significant discrepancy observed in eGFR values between patients with complicated and uncomplicated plaques (*p* = 0.004), and considering low GFRs’ implication in intraplaque hemorrhage (*p* = 0.012), comprehensive analysis was conducted on both personal data and pathological history, respectively, in order to elucidate the histological and biological variables that distinguished the subgroups A–C.

Between women and men, eGFR showed a statistically significant difference (*p* = 0.041); otherwise, eGFR values did not correlate with the listed comorbidities (*p* > 0.05).

When we analyzed the distribution of complicated and non-complicated plaques between the kidney function subgroups (A–C), we observed a strong association between low eGFR and destabilizing factors, resulting in plaque complications. In patients with an eGFR < 60 mL/min/1.73 m^2^ (9/9), all-cause complicated carotid plaques were observed in all cases (100%). The early-stage kidney disease (group B) was associated with plaque complications in 26.8% (11/41) of cases. In the subgroup with normal kidney function, the incidence of plaque complications was the lowest (8/41), representing only 19.5% of the total cases, and these differences across the groups were significant (*p* = 0.003). In a logistic regression model, an eGFR above 85 mL/min/1.73 m^2^ was found to be a significant predictor of histological plaque complications when adjusted for age and diabetes (Supplementary Table S2).

**Figure 4:**
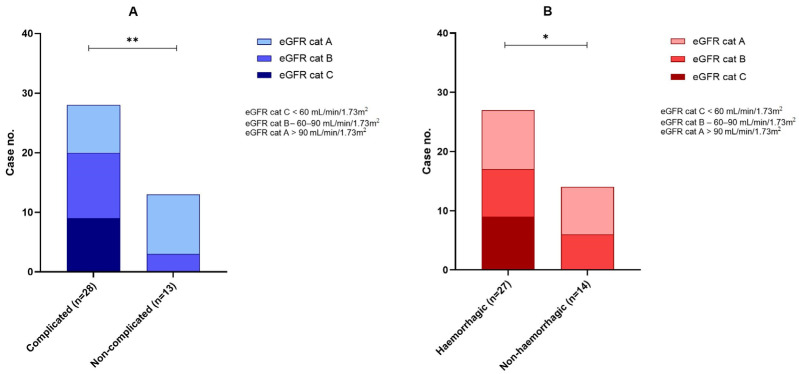
All-cause (sub-figure (**A**)) and hemorrhagic (sub-figure (**B**)) complications in eGFR A, B, and C categories. * *p* < 0.05, ** *p* < 0.01.

This association also held for intraplaque hemorrhage, which was present in all renal failure cases (21.9% of the total). The percentage of this plaque instability factor decreased sharply in groups B (57.14%) and C (55.55%); patients with renal failure are prone to plaque destabilization by intraplaque hemorrhage (*p* = 0.012) (Table 4, Figure 4).

## 4. Discussion

Carotid atherosclerosis was found to be a significant contributor to cerebrovascular events, particularly strokes, and its management often involves surgical intervention such as carotid endarterectomy (CEA). This procedure has been established as the gold standard for treating symptomatic high-grade stenosis of the internal carotid artery, especially in patients with chronic kidney disease (CKD) [29,30]. The relationship between carotid atherosclerosis and CKD is critical, as patients with CKD are at increased risk for cardiovascular events, including stroke, due to the accelerated atherosclerotic process [31].

The efficacy of CEA in patients with CKD has been supported by various studies, indicating that it significantly reduces the risk of ipsilateral stroke in those with symptomatic high-grade stenosis [29,30]. Specifically, a study analyzing data from the North American Symptomatic Carotid Endarterectomy Trial found that patients with stage 3 CKD who underwent CEA saw a notable reduction in stroke risk compared to those receiving medical management alone [29]. Furthermore, the durability of stroke prevention following CEA has been well-documented, with long-term follow-ups showing sustained benefits in stroke risk reduction [30,32].

To achieve our study objectives, we analyzed the classic features of plaque morphology (inflammatory mononuclear infiltrate, necrotic lipid core, micro- and macrocalcification, ulceration, thrombosis, hemorrhage, and neoangiogenesis) and measured the intensity of intraplaque C-reactive protein and MMP-9 expression using two different digital immunohistology tools. In addition, we determined the serum concentrations of MCP-1, oxLDL, CRP, and MMP-9 in each case to assess the relationship between peripheral inflammatory biomarkers, inflammatory indices derived from CBC, and the histological findings in the carotid plaque removed by the CEA procedure. Also, the study aimed to correlate the renal function with biological and histological indicators of plaque vulnerability. It is important to note that our endarterectomy specimens represented advanced stages of carotid stenosis, with significant mononuclear cell infiltrate and late-stage calcification in almost 3/4 of the cases, and a necrotic lipid core in 44% of the cases. The most frequent histological complications were intraplaque hemorrhage and neovascularization; less commonly, we saw thrombosis or ulceration.

Elevated levels of hsCRP, a marker of systemic inflammation, are linked to both CKD and cardiovascular diseases, indicating that chronic inflammation may exacerbate atherosclerotic processes in these patients [33]. The relationship between MCP-1 and carotid atherosclerosis is particularly noteworthy, as systemic inflammation in CKD can lead to oxidative stress and endothelial dysfunction, both of which are critical in the pathogenesis of atherosclerosis [34]. Furthermore, the presence of elevated MCP-1 levels has been associated with increased carotid intima media thickness (IMT), a well-established indicator of atherosclerosis [34,35]. However, some authors confirmed the relationship with IMT in stroke-free participants but failed to reveal associations of MCP-1 levels with atherosclerotic plaque morphology [36]. The inflammatory milieu in CKD, characterized by elevated MCP-1, exacerbates the progression of carotid atherosclerosis, highlighting the need for targeted therapies that address these inflammatory pathways [37].

By corroborating histological data with peripheral parameters, our study revealed that for patients with at least one plaque complication, the hsCRP levels were higher in the all-complications-positive (COMP+) group, while MCP-1 and MMP-9 plasma levels were lower in the COMP+ group, without statistical significance, suggesting that changes in the vascular intima, at least in our cohort, might not be mirrored in the periphery. However, these results could be affected by the relatively reduced number of cases included in our cohort. In contrast to the above findings, our cohort showed lower circulating MCP-1 levels in the subgroup with an inflammatory infiltrate. This bias could be influenced by the late stage of plaque development, as reflected by the high grade of stenosis and the almost generalized macrocalcification. The role of MCP-1 remains questionable. Georgakis et al. observed a significantly increased presence of MCP-1 in 1199 patients exhibiting various signs of plaque vulnerability, including a large lipid core, low collagen content, a high macrophage burden, a low smooth muscle cell burden, and intraplaque hemorrhage. However, the authors were unable to establish a correlation between MCP-1 protein quantities isolated from plaques and those determined from sera. They concluded that circulating MCP-1 levels may not be a marker of plaque MCP-1 [38].

The involvement of matrix metalloproteinase-9 (MMP-9) in atherosclerotic plaque destabilization is critical. Research indicates that MMP-9 is abundantly expressed in atherosclerotic plaques, particularly in areas associated with inflammation. Specifically, MMP-9 is predominantly produced by M2 macrophages, which are crucial for the inflammation resolution in atherosclerotic lesions, suggesting that MMP-9 activity is closely linked to macrophage phenotype dynamics [39]. Matrix metalloproteinase 9 (MMP 9) may contribute to plaque formation and progression since it is a zinc-dependent endopeptidase that facilitates the breakdown of extracellular matrix components. Elevated levels of MMP-9 are associated with the remodeling of both lipid-rich areas and fibrous caps within plaques, rendering them susceptible to rupture and leading to acute ischemic events [40]. There are discrepancies in reports of MMP-9 expression, with some studies showing a significantly elevation in asymptomatic, but not in symptomatic regions [8]; however, Sef et al. found a higher expression of MMP-9 in unstable carotid plaque and symptomatic patients’ endarterectomy specimens [41]. The relationship between serum MMP 9 levels and the tissue expression of MMP 9 in carotid plaques might be of specific interest when assessing plaque vulnerability and predicting the risk of atherothrombotic stroke [42]. Research indicates that MMP-9 levels are notably higher in asymptomatic individuals than those with symptomatic plaques, suggesting a protective mechanism in the former group [8], but there are also divergent observations [43]. Our study could not establish clear relationships, as MMP-9 plasma levels were slightly higher in patients without complications, without reaching the level of statistical significance.

The multifaceted role of MMP-9 in both circulating blood as well as within the plaque microenvironment emphasizes its relevance in understanding atherosclerotic disease progression and highlights its potential utility in therapeutic targeting [44].

Elevated serum oxLDL levels are directly correlated with the development and progression of atherosclerotic plaques, particularly within the carotid arteries. This relationship is attributed to oxLDL’s facilitation of inflammatory processes and foam cell formation, which are essential in plaque dynamics. Specifically, oxLDL interacts with scavenger receptors on macrophages, promoting their uptake and leading to the formation of lipid-laden foam cells. Furthermore, oxLDL drives inflammation by releasing pro-inflammatory cytokines that exacerbate atherogenesis. As emerging studies emphasize the need for targeted therapeutic strategies, the transition towards understanding the role of oxLDL offers a promising avenue for addressing atherosclerotic pathologies in clinical settings [45,46].

Previous research highlighted that patients exhibiting unstable plaques had notably elevated oxLDL levels, with a cutoff point of oxLDL >31.4 ng/mL showing an 82.5% probability of instability, positioning oxLDL as a potential independent marker for assessing plaque conditions [47]. In our study, serum levels of oxLDL were found to be higher in all patients, with a median of 51.89 (17.59–234.21) mg/dl. Although oxLDL was found to be higher in the COMP+ compared to the COMP− group, its did not reach the threshold of statistical significance.

The neutrophil-to-lymphocyte ratio and the lymphocyte-to-monocyte ratio, which are indicative of systemic inflammation, have also been linked to atherosclerosis progression [48,49,50,51]. Elevated NLR has been associated with increased carotid intima media thickness and the presence of carotid plaques, suggesting that neutrophils and lymphocytes contribute to the inflammatory milieu that promotes atherosclerosis [52].

Based on the results of our previous study, in which NLR, PLR, and LMR modifications were predictors for the amputation rate in PAD patients who underwent a revascularization procedure [53], in this study, we also investigated the relationship of these markers with histologic signs of plaque vulnerability, complementing our analyses with SIRI and NLR.

Although median LMR was within the reference range, the median NLR values were in the gray zone, which may represent an early warning of a pathological condition or process such as atherosclerosis [54]. Furthermore, monocytes are prone to infiltrate atherosclerotic plaques, even though their values are not higher than normal [55]. However, our results did not reveal differences in these parameters between patients with/without complicated atherosclerotic plaque.

There is increasing evidence that the progression of chronic kidney disease contributes to an increased risk of cerebrovascular disease [56,57]. Not all the interactions are clear; there may be several mechanisms that promote plaque progression through interrelated biological systemic changes based on kidney disease. Pelisek et al. compared histologically CKD and non-CKD carotid atherosclerosis groups and described significantly more micro- and macrocalcifications, as well as more unstable and ruptured plaques, in those with CKD. In addition, serum samples from CKD patients showed higher levels of inflammatory molecules, such as C-reactive protein and fibrinogen, parathyroid hormone and metalloproteinase MMP-7, but not of MMP-9 [58]. Numerous abnormalities of the coagulation and fibrinolytic systems have been reported in a CKD context. In addition to plasma fibrinogen, circulating levels of factors VII and VIII and the von Willebrand factor, as well as prothrombin fragment 1.2 and thrombin–antithrombin complex, are high, suggesting a hypercoagulable state [59]. Observational studies have described elevated factor XIII antigen, activity, tissue plasminogen activator, plasminogen activator inhibitor-1, D-dimer, and low alpha-2 plasmin inhibitor [60,61,62].

The Rotterdam Study performed by Selwaness et al. found that both carotid artery stenosis and intraplaque hemorrhage were predictive of ischemic stroke in men, but not in women [63]. Increased neovascularization and intraplaque hemorrhage of coronary artery lesions, associated with the local accumulation of oxLDL and low GFR (<30 mL/min/1.73 m^2^), were reported in elderly Japanese patients by Nakano et al. [64]. In a subgroup of SPRINT (Systolic Blood Pressure Intervention Trial) participants assessed by carotid MRI at baseline and 2.5 years, chronic kidney disease was associated with lower odds of necrotic core growth, and the presence of necrotic core, but not thickness, was predictive of cardiovascular events [65]. Wesseling et al. studied 1796 carotid endarterectomy specimens and found a higher odds ratio for intraplaque hemorrhage in those with poor renal function [66]. The authors further stated that local inflammation does not confer a risk of vulnerability and poor cardiovascular outcomes in CKD patients.

The most relevant finding in our study was that the reduced eGFR, an indicator of impaired renal function, was significantly lower in patients with complicated plaque (any of hemorrhage, neovascularization, ulceration, or thrombosis) (*p* = 0.004). A relatively large study analyzing kidney function and intraplaque complications in patients who underwent CEA found that the impairment of renal function was best correlated with plaque hemorrhage but not with inflammatory features of the atherosclerotic plaque. The same author stated that decreasing renal function by 20 mL/min/1.73 m^2^ was associated with an increased risk of intraplaque hemorrhage and fibrous atheroma. The mechanisms beyond inflammation that might be involved in plaque vulnerability were supposed to be upregulation of the classical complement pathway and the intrinsic coagulation pathway [67].

In our study, we observed a strong association between low eGFR and destabilizing factors that result in plaque complications. In patients with impaired renal function and eGFR < 60 mL/min/1.73 m^2^, complicated carotid plaques were observed in all cases, while complications were recorded in 78.5% of cases involving mild kidney insufficiency. In the subgroup with normal kidney function, the incidence of plaque complications was the lowest, representing only 44.4% of the total (*p* = 0.002). These findings underscore the impact of the reduced eGFR on intraplaque complications in patients who underwent CEA with CKD, highlighting the need for careful assessment and management of cardiovascular risk in the CKD population.

There were several limitations in our study. Only a limited number of subjects were included in the study’s implementation stage. Stringent selection criteria regarding clinical and laboratory data and the quantity and quality of the processed material (calcification, large necrotic lipid core, intraplaque hemorrhage, etc.) led to the exclusion of a relatively large number of cases. Another problem was the fragmentation of the material after endarterectomy, which made it difficult to interpret the overall histological changes and to accurately quantify the antigen under investigation. However, we believe that this study provides additional information due to the complexity of the methodology, as there are relatively few works investigating circulating biomarkers in parallel with those formed in situ at the atherosclerotic plaque.

## 5. Conclusions

Signs of plaque vulnerability, particularly intraplaque hemorrhage, were observed to be associated with reduced renal function. Patients with intraplaque hemorrhage and ulceration from the carotid plaque had a higher neutrophil/lymphocyte ratio. Significantly higher expression of MCP-1 was identified in plaques with massive inflammatory infiltrate. However, these findings do not appear to be related to circulating levels of hsCRP, MMP-9, or oxLDL in patients with advanced carotid artery disease. The study’s findings underscore the critical need for meticulous cardiovascular risk assessment in patients with CKD, particularly those with severe carotid stenosis, highlighting the profound association between impaired renal function and plaque complications.

## Figures and Tables

**Table 1:** Baseline characteristics of the study population.

Variable	*Median* (Min–Max) or Frequency *n* (%)
***Demographic and lifestyle variables***	
Age (years)	67/(42–80)
Gender (f/m)	12 (29.27)/29 (70.73)
Smoking (yes/no)	16 (39.02)/25 (60.98)
***Disease characteristics and comorbidities***	
Grade of stenosis (%)	83.47 (70–95)
Stroke history (y/n)	26 (63.41)/15 (36.5)
Hypertension (y/n)	39 (95.12)/2 (4.88)
Diabetes (y/n)	15 (36.59)/26 (63.41)
Polyvascular disease (2 or 3/1 arterial beds affected)	36 (87.8)/5 (12.2)
Ischemic heart disease (y/n)	34 (82.92)/7 (17.08)
Chronic kidney disease (CKD) (y/n)	9 (21.95)/32 (78.04)
eGFR	85 (24–114)
GFR-GROUP A	95.5 (90–114)
GFR-GROUP B	78 (61–87)
GFR-GROUP C	43 (24–58)
Body mass index	28.37 (20–47.26)
***Biological variables***	
Dyslipidemia (yes/no)	38 (92.68)/3 (7.32)
Abs. neutrophil count (×10^3^/µL)	5.16 (2.4–9.95)
Abs. lymphocyte count (×10^3^/µL)	1.93 (0.82–5.27)
Abs. monocyte count (×10^3^/µL)	0.62 (0.36–1.09)
Neutrophil-to-lymphocyte ratio (NLR)	2.4 (1.1–5.02)
Lymphocyte-to-monocyte ratio (LMR)	3.21 (1.77–7.98)
Systemic Inflammation Response Index (SIRI)	1.59 (0.59–5.48)
High-sensitivity C-reactive protein (hsCRP), (mg/l)	1.01 (0.16–29.2)
Monocyte chemoattractant protein-1 (MCP-1) (pg/mL)	193.67 (75.97–847.06)
Matrix metalloproteinase-9 (MMP-9) (ng/mL)	67.98 (1.94–452.5)
Oxidized low-density lipoprotein (ng/mL)	51.89 (17.59–234.51)
***Signs of plaque vulnerability***	
Inflammatory infiltrate	32 (78.1)
Macro-/microcalcification	30 (73.2)
Lipid core	18 (43.9)
Neovascularization	35 (85.4)
Hemorrhage	27 (65.8)
Thrombosis	4 (17.1)
Ulceration	4 (9.7)

Values are shown as median (min–max range) for undivided parameters and as absolute numbers (percentages) for grouped parameters. The words in bold represent the type of parameters from the columns and the words in bold and italics represent the group of parameters.

**Table 2:** Clinical, biological, and histological characteristics associated with histological evidence of complicated plaques.

Variable	COMPL+ Group (*n* = 28)	COMPL− Group (*n* = 13)	*p* Values
***Demographic and lifestyle variables***			
Age (years)	66.5 (42–80)	67 (57–78)	0.970
BMI	28.28 (21.22–47.26)	29.39 (20–35.06)	0.533
Smoking (yes/no)	11 (26.82)/17 (41.46)	4 (9.75)/9 (21.95)	0.434
***Disease characteristics and comorbidities***			
Stroke history (y/n)	18 (43.9)/10 (24.39)	8 (19.51)/5 (12.19)	0.615
Hypertension (y/n)	26 (63.41)/2 (4.87)	13 (31.7)/0	0.307
Diabetes (y/n)	8 (19.51)/20 (48.78)	7 (17.07)/6 (14.63)	0.224
Polyvascular disease (2 or 3/1 arterial beds affected)	25 (60.97)/3 (7.31)	10 (24.39)/3(7.31)	0.276
***Plaque charcteristics***			
Inflammation (y/n)	23 (82.14)/5 (17.85)	9 (69.23)/4 (30.76)	0.428
Microcalcification (y/n)	3 (10.71)/25 (89.28)	2 (15.38)/11 (84.61)	0.643
CRP + surface	67.04 (45.9–76.92)	66.04 (45.71–75.69)	0.857
CRP-H-score	16.11 (1–128.24)	5.12 (1.41–93.08)	0.095
MMP-9 + surface	62.89 (48.67–75.76)	60.93 (51.12–73.45)	0.310
MMP-9-H-score	32.63 (4.27–141.17)	35.02(3.8–135.8)	0.608
***Biological variables***			
Abs. neutrophil count (×10^3^/µL)	5.33 (2.4–9.95)	4.93(2.82–7.59)	0.729
Abs. lymphocyte count (×10^3^/µL)	1.83 (1.01–5.27)	2.09 (0.82–3.43)	0.814
Abs. monocyte count (×10^3^/µL)	0.625 (0.37–0.48)	0.59 (0.36–1.08)	0.708
Neutrophil-to-lymphocyte ratio (NLR)	2.48(1.1–5.02)	2.18 (1.65–4.56)	0.688
Lymphocyte-to-monocyte ratio (LMR)	3.51(1.77–7.98)	3.04 (2.28–6.23)	0.857
eGFR	79.5 (24–110)	94 (69–114)	0.004
Systemic Inflammation Response Index (SIRI)	1.59 (0.59–5.48)	1.45 (0.63–2.69)	0.750
High-sensitivity C-reactive protein (hsCRP) (mg/L)	2.17 (0.16–29.2)	0.7 (0.16–22.0)	0.167
Monocyte chemoattractant protein-1 (MCP-1) (pg/mL)	181.94 (75.97–847.06)	228.57 (151.18–626.89)	0.135
Matrix metalloproteinase-9 (MMP-9) (ng/mL)	62.38 (1.94–452.5)	85.87 (33.24–249.48)	0.167
Oxidized low-density lipoprotein (ng/mL)	53.98 (17.59–234.21)	46.12 (27.03–68.95)	0.445

Values of variables with abnormal distribution are shown as median (min–max). Binomial variables are represented as absolute numbers and percentages (in brackets). Comparison of variables with discrete values was performed by Fisher’s exact test (2 × 2 groups) and Pearson χ^2^ test (3 × 2 groups). For numeric variables, groups were compared with the Mann–Whitney U test. Level of statistical significance set to *p* = 0.05. COMPL = complications (intraplaque hemorrhage, neovascularization, endothelial surface ulceration with or without thrombosis). The words in bold represent the type of parameters from the columns and the words in bold and italics represent the group of parameters.

**Table 3:** Laboratory parameters and serum biomarkers in patients with and without inflammation of carotid plaque.

Laboratory Parameters	Patients Without Inflammation (*n* = 9)	Patients with Inflammation (*n* = 32)	*p*-Value
Abs. neutrophil count (×10^3^/µL)	5.93 (5.5–6.46)	4.82 (4.05–5.82)	0.144
Abs. lymphocyte count (×10^3^/µL)	1.56 (1.46–2.95)	1.94 (1.65–2.73)	0.362
Neutrophil-to-lymphocyte ratio (NLR)	3.05 (2.18–4.06)	2.39 (1.75–3.13)	0.384
Abs. monocyte count (×10^3^/µL)	0.59 (0.49–0.76)	0.63 (0.48–0.82)	0.718
Lymphocyte-to-monocyte ratio (LMR)	3.04 (2.64–3.95)	3.59 (2.74–4.12)	0.362
Systemic Inflammation Response Index (SIRI)	1.87 (1.14–2.36)	1.54 (1.09–1.85)	0.362
Creatinine (mg/dL)	0.9 (0.88–1.12)	0.98 (0.82–1.13)	0.659
High-sensitivity C-reactive protein (hs-CRP) (mg/L)	1.01 (0.54–6.32)	1.06 (0.55–5.89)	0.936
Monocyte chemoattractant protein-1 (MCP-1) (pg/mL)	254.77 (204.95 –309.87)	177.99 (151.30–231.97)	0.01
Matrix metalloproteinase-9 (MMP-9) (ng/mL)	59.37 (47.89–86.89)	71.02 (41.34–101.90)	0.952
Oxidized low-density lipoprotein (oxLDL) (ng/mL)	51.73 (41.09–55.53)	52.16 (33.61–66.5)	0.887

Values of variables with abnormal distribution are shown as median (min–max range). Groups were compared with Mann–Whitney U test. Level of statistical significance set to *p* = 0.05. The words in bold represent the type of parameters from the columns and the words in bold and italics represent the group of parameters.

**Table 4:** Distribution of complications (all) and specific intraplaque hemorrhage among different categories of kidney dysfunction evaluated by eGFR.

eGFR	COMPL− Group (*n* = 13)	COMPL+ Group (*n* = 28)	IPH− Group (*n* = 14)	IPH+ Group (*n* = 27)
C	0 (0)	9 (21.9)	0 (0)	9 (21.9)
B	3 (7.3)	11 (26.8)	6 (14.6)	8 (19.5)
A	10 (24.4)	8 (19.5)	8 (19.5)	10 (24.4)

Values are shown as case numbers/percentage of all in brackets. The words in bold represent the type of parameters from the columns and the words in bold and italics represent the group of parameters.

## Data Availability

Data spreadsheets available as Balmos, Ioan (2025), “Database Article Life Journal—Balmos Ioan Alexandru”, Mendeley Data, V1, https://doi.org/10.17632/3m5rghpx49.1; https://data.mendeley.com/preview/3m5rghpx49?a=93b0c64f-01c7-4b3f-b658-7a1c51abbc38 (accessed on 29 April 2025).

## Supplementary Materials

The following supporting information can be downloaded at: https://www.mdpi.com/article/10.3390/life15071008/s1, Table S1: Coefficient of significance for the association of biological changes with different signs of plaque vulnerability, Table S2: Logistic regression model for the prediction of any-cause plaque complications.

## Abbreviations

The following abbreviations are used in this manuscript:

CKD	Chronic kidney disease
CEA	Carotid endarterectomy
CBC	Complete blood count
eGFR	Glomerular filtration rate
NLR	Neutrophil/lymphocyte ratio
LMR	Lymphocyte/monocyte ratio
SIRI	Systemic inflammatory response index
MMP-9	Matrix metalloproteinase-9
MCP-1	Monocyte chemoattractant protein-1
oxLDL	Oxidized forms of human low-density lipoprotein
hsCRP	High-sensitivity C-reactive protein
H&E	Hematoxylin and eosin
IHC	Immunohistochemistry
