# Supplementary material for: The Association of Histological Signs of Plaque Instability with Low eGFR, Higher Neutrophil-to-Lymphocyte Ratio, and Lower Serum MCP-1 Levels in Carotid Endarterectomy Patients—A Single-Center, Prospective Cohort Study
Source: Life (Basel). 2025 Jun 25;15(7):1008. doi: 10.3390/life15071008 (PMC12298409; doi:10.3390/life15071008)
Supplement: Supplementary file 1 [file life-15-01008-s001.zip › Supplementary Tables S1 and S2 Final/Table S1 Article Balmos Ioan Alexandru.pdf]

Table S1. Coefficient of significance for the association of biological changes with different signs of plaque vulnerability

**Plaque characteristics**	**MC$^{+/-}$ (5/36)**	**INF$^{+/-}$ (32/9)**	**ULC$^{+/-}$ (4/37)**	**OM$^{+/-}$ (16/25)**	**NV$^{+/-}$ (35/6)**	**THR$^{+/-}$ (7/34)**	**LLC$^{+/-}$ (18/23)**	**IPH$^{+/-}$ (14/27)**
	***p*-value**	***p*-value**	***p*-value**	***p*-value**	***p*-value**	***p*-value**	***p*-value**	***p*-value**
eGFR (A)	0.222	0.998	0.325	0.554	0.925	0.256	0.451	0.012
Abs. neutrophil count (×10³/μL)	1.015	0.145	0.082	0.606	0.252	0.696	0.458	0.203
Abs. lymphocyte count (×10³/μL)	0.202	0.359	0.203	0.905	0.086	0.622	0.004	0.194
NLR	0.360	0.392	0.025	0.989	0.061	0.186	0.125	0.039
Abs. monocyte count (x10³/μL)	0.448	0.721	0.336	0.587	0.222	0.696	0.224	0.505
LMR	0.892	0.675	0.750	0.662	0.601	0.852	0.412	0.881
SIRI	0.923	0.359	0.172	0.701	0.268	0.385	0.687	0.257
hsCRP	0.548	0.938	0.510	0.282	0.732	0.224	0.687	0.558
MCP-1	0.831	0.008	0.566	0.434	0.481	0.799	0.315	0.293
MMP-9	0.497	0.938	0.596	0.112	0.301	0.164	0.507	0.615
oxLDL (ng/mL)	0.301	0.889	0.983	0.226	0.552	0.773	0.845	0.881
CRP_positive surface	0.772	0.359	0.082	0.042	0.899	0.773	0.315	0.577
CRP-H-score	0.301	0.392	0.145	0.761	0.375	0.986	0.354	0.055
MMP-9 _positive surface	0.574	0.163	0.019	0.404	0.159	0.385	0.214	0.121
MMP-9-H-score	0.628	0.183	0.406	0.075	0.957	0.799	0.205	0.281

MC-microcalcification; INF-intraplaque inflammatory infiltrate; ULC-ulceration; OM-osteoid metaplasia NV-neovascularisation; THR-atherothrombosis; LLC-large lipid core; IPH-intraplaque hemorrhage. The words in bold represent the type of parameters from the columns and the words in bold and italics represent the group of parameters.
