# Supplementary material for: The Association of Histological Signs of Plaque Instability with Low eGFR, Higher Neutrophil-to-Lymphocyte Ratio, and Lower Serum MCP-1 Levels in Carotid Endarterectomy Patients—A Single-Center, Prospective Cohort Study
Source: Life (Basel). 2025 Jun 25;15(7):1008. doi: 10.3390/life15071008 (PMC12298409; doi:10.3390/life15071008)
Supplement: Supplementary file 1 [file life-15-01008-s001.zip › Supplementary Tables S1 and S2 Final/Table S2 Article Balmos Ioan Alexandru.pdf]

Table S2. Logistic regression model for the prediction of any-cause plaque complications

**Variable**	**Estimate (*β*)**	**OR (95% CI)**	***p*-value**
Age (>67 years)	-0.25	0.78 (0.18 – 3.36)	0.736
Diabetes (yes vs. no)	-1.16	0.31 (0.06 – 1.56)	0.159
eGFR <85 mL/min/1.73m^2	-1.92	0.15 (0.03 – 0.73)	0.027

The level of statistical significance has been set to p=0.05, The words in bold represent the type of parameters from the columns and the words in bold and italics represent the group of parameters.
